# Response diversity in Mediterranean coralligenous assemblages facing climate change: Insights from a multispecific thermotolerance experiment

**DOI:** 10.1002/ece3.5045

**Published:** 2019-03-12

**Authors:** Daniel Gómez‐Gras, Cristina Linares, Sonia de Caralt, Emma Cebrian, Maša Frleta‐Valić, Ignasi Montero‐Serra, Marta Pagès‐Escolà, Paula López‐Sendino, Joaquim Garrabou

**Affiliations:** ^1^ Departament de Biologia Marina Institut de Ciències del Mar (CSIC) Barcelona Spain; ^2^ Departament de Biologia Evolutiva, Ecologia i Ciències Ambientals, Institut de Recerca de la Biodiversitat (IRBIO) Universitat de Barcelona Barcelona Spain; ^3^ Centre d' Estudis Avançats de Blanes (CSIC) Blanes Spain; ^4^ GR MAR, Institut d'Ecologia Aquàtica, Facultat de Ciències Universitat de Girona Girona Spain

**Keywords:** Benthic communities, corals, ocean warming, sponges, temperate reefs, thermotolerance

## Abstract

Climate change threatens coastal benthic communities on a global scale. However, the potential effects of ongoing warming on mesophotic temperate reefs at the community level remain poorly understood. Investigating how different members of these communities will respond to the future expected environmental conditions is, therefore, key to anticipating their future trajectories and developing specific management and conservation strategies. Here, we examined the responses of some of the main components of the highly diverse Mediterranean coralligenous assemblages to thermal stress. We performed thermotolerance experiments with different temperature treatments (from 26 to 29°C) with 10 species from different phyla (three anthozoans, six sponges and one ascidian) and different structural roles. Overall, we observed species‐specific contrasting responses to warming regardless of phyla or growth form. Moreover, the responses ranged from highly resistant species to sensitive species and were mostly in agreement with previous field observations from mass mortality events (MMEs) linked to Mediterranean marine heat waves. Our results unravel the diversity of responses to warming in coralligenous outcrops and suggest the presence of potential winners and losers in the face of climate change. Finally, this study highlights the importance of accounting for species‐specific vulnerabilities and response diversity when forecasting the future trajectories of temperate benthic communities in a warming ocean.

## 1 INTRODUCTION

From polar oceans to tropical seas, climate change dramatically affects marine ecosystems by influencing processes at all levels of biological organization (Doney et al., 2012; Poloczanska et al., 2016; Scheffers et al., 2016). Moreover, this anthropogenic pressure will continue to cause unprecedented impacts in the oceans during the next decades as global sea surface temperatures continue to rise and marine heat waves become more frequent and intense (Bellard, Bertelsmeier, Leadley, Thuiller, & Courchamp, 2012; Oliver et al., 2018). However, climate change effects have contrasting impacts on biotas (McKinney & Lockwood, 1999). Therefore, understanding how different species, populations and communities will respond to warming is key to developing specific conservation and management strategies aimed at enhancing the resilience of vulnerable marine ecosystems.

Coastal benthic communities such as tropical and temperate reefs are among the most biologically diverse and socioeconomically valuable systems on the planet (Ballesteros, 2006; Bennett et al., 2016; Spalding, Ravilious, & Green, 2001). Nonetheless, when facing global warming, they are especially under threat. As migrating toward more thermally suitable conditions is not an option for most sessile species, most organisms from these communities will be compelled to rely on effective acclimatization (an adjustment of physiology via phenotypic plasticity) or adaptation (an increased abundance of tolerant genotypes over generations) processes to prevail. Although these two mechanisms that evolved for coping with environmental change will likely allow diverse species and/or populations to persist (Palumbi, Barshis, Traylor‐Knowles, & Bay, 2014), increasing evidence indicates that the unusually high rates of warming and the increasing frequency of extreme events may prevent many others from effectively doing so (Heron et al., 2017; Hoegh‐Guldberg, Poloczanska, Skirving, & Dove, 2017; Hughes et al., 2017, 2018). In this situation, it is likely that as temperatures continue to rise species with lower thermal thresholds will more frequently be exposed to temperatures beyond their tolerance limits (especially during marine heat waves), potentially hindering adaption/acclimatization processes and favoring responses that range from sublethal effects to death and local extinction (Somero, 2010). The likely loss of such sensitive species would not only change the composition of benthic communities but also diminish the functions and services that they provide. However, if there is response diversity among functionally redundant organisms, the insurance hypothesis of biodiversity suggests that the overall ecosystem functionality may be stabilized through compensatory dynamics among species (Gonzalez & Loreau, 2009; Mori, Furukawa, & Sasaki, 2013; Yachi & Loreau, 1999). Exploring species‐specific thermal sensitivities among different components of benthic communities is, therefore, a key step toward forecasting the future composition and functionality of these communities in the face of climate change. However, while important efforts in this direction have been taken in shallow tropical reefs, thermotolerance analyses in temperate benthic communities largely lag behind (Kersting et al., 2015; Linares, Cebrian, Kipson, & Garrabou, 2013; Savva, Bennett, Roca, Jordà, & Marbà, 2018; Torrents, Tambuté, Caminiti, & Garrabou, 2008).

In the Mediterranean, coralligenous assemblages are one of the most affected habitats by climate change. Coralligenous assemblages are biogenic formations built by the growth of crustose coralline algae and diverse calcareous macroinvertebrates at low irradiance levels and are characterized by their great structural complexity and species richness (harbouring ~10% of marine Mediterranean species) (Ballesteros, 2006). Most of the structural species of these habitats exhibit slow population dynamics and long life spans (+100 years; Garrabou & Harmelin, 2002; Linares, Doak, Coma, Diaz, & Zabala, 2007; Teixidó, Garrabou, & Harmelin, 2011); therefore, they are very sensitive to disturbances, including climate change (Balata, Piazzi, & Benedetti‐Cecchi, 2007; Ferrigno, Appolloni, Russo, & Sandulli, 2018; Garrabou et al., 2009; Montero‐Serra et al., 2015). In fact, more than 30 coralligenous species from different phyla and different structural roles have been affected in various mass mortality events (hereafter MMEs) associated with Mediterranean heat waves, suffering extensive tissue necrosis (partial and total mortality) and long‐term population declines (Cerrano et al., 2000; Crisci, Bensoussan, Romano, & Garrabou, 2011; Garrabou et al., 2009; Garrabou, Perez, Sartoretto, & Harmelin, 2001; Linares et al., 2005). Moreover, for some key habitat‐forming species, these population declines have been shown to potentially drive detrimental effects at the community level, such as the reduction of structural complexity and resilience (Linares et al., 2017; Ponti et al., 2014). However, while some species have been massively and recurrently affected during these warming events, other taxonomically and morpho‐functionally related organisms seem to remain unaffected, triggering the question of whether there could be different levels of thermal sensitivity within these communities in the context of climate change. This could have further implications for the future composition of these habitats and the loss (or maintenance) of the many associated functions and services they provide.

In this study, we experimentally assessed the thermal response of 10 abundant, representative and widely distributed species from these communities that belong to different phyla and encompass contrasting growth forms. The main aim was to explore whether co‐occurring species of these highly diverse habitats differ in their thermal sensitivities, as field observations suggest, in view to discuss the implications of climate change on the composition and functioning of these key Mediterranean habitats. Our results contribute to filling the gap of thermotolerance data for coralligenous assemblages and suggest the presence of potential winners and losers in the face of ocean warming.

## 2 MATERIALS AND METHODS

### 2.1 Model species

We used a total of 10 abundant and representative species from three different phyla (cnidaria, porifera, and tunicata) and four different growth forms (including encrusting, massive, cup and/or tree‐like forms) that are commonly and ubiquitously found in Mediterranean coralligenous assemblages over the whole Mediterranean basin (Ballesteros, 2006; Casas‐Güell, Teixidó, Garrabou, & Cebrian, 2015). Specifically, we used three species of anthozoans (cnidarians): *Parazoanthus axinellae*(Schmidt, 1862)*, Leptopsammia pruvoti*(de Lacaze‐Duthiers, 1897), and *Alcyonium acaule* (Marion, 1878); six species of demosponges (poriferans): *Agelas oroides*(Schmidt, 1864), *Axinella polypoides* (Schmidt, 1862), *Axinella damicornis* (Esper, 2784), *Crambe crambe* (Schmidt, 1862), *Dysidea avara* (Schmidt, 1862), and* Petrosia ficiformis* (Poiret, 1789); and one species of ascidian (tunicate): *Cystodytes dellechiajei*(Della Valle, 1877) (See Supporting information Methods S1, Figure S1 and Table S1). For *Parazoanthus axinellae*, two different and easily distinguished morphotypes were used: the rather yellow and thin “slender” morphotype, which mostly lives in rocky substrates but can also usually be found as an epibiont on demosponges, and the bright orange “stocky” morphotype, which is mainly found in primary substrates (Cachet et al., 2015). We distinguished between these two morphotypes because of the ongoing scientific debate about whether they could, in fact, be two separated species (Cachet et al., 2015). Moreover, the presence of highly bioactive secondary metabolites, named “parazoanthines,” in only the “slender” morphotype could potentially lead to contrasting responses to warming between these two morphotypes, as secondary metabolites are usually associated in plants and many benthic organisms with protection against different abiotic and biotic stresses, including warming (Bennett & Wallsgrove, 1994; Cachet et al., 2015; Reverter, Perez, Ereskovsky, & Banaigs, 2016).

### 2.2 Review on mass mortalities of the selected species

We reviewed all information available in the scientific literature regarding warming‐ induced MMEs reported on any of the 10 selected species that occurred in the North‐Western (NW) Mediterranean from 1983 to 2017. In particular, we followed the same methodology described by Rivetti, Fraschetti, Lionello, Zambianchi, and Boero (2014) and Marbà et al. (2015) and expanded the search to 2017. From this combined search, we only selected articles that reported the mass mortality of any of the 10 selected species, referred to the NW Mediterranean and in which the mortality was directly attributed to warming. The resulting information regarding dates, locations, depths, species affected, identified cause, and references can be found in Supporting information Table S2. This information allowed us to investigate whether experimental species‐specific thermal sensitivities were concomitant to the level of vulnerability shown in the field by the different selected species during previous MMEs.

### 2.3 Sampling and biological material

**Figure 1:**
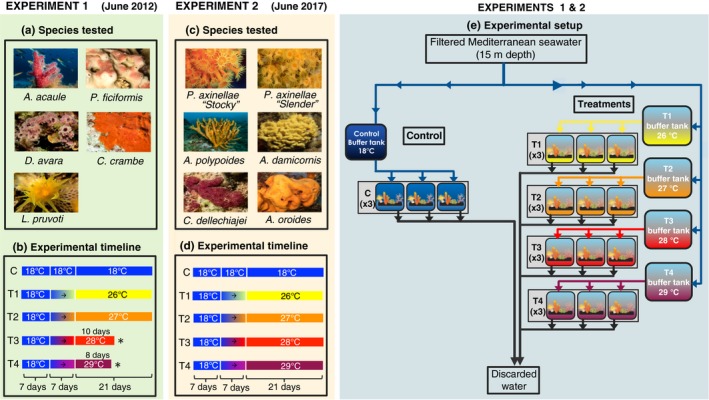
Species tested (a, b), experimental timeline (c, d), and experimental setting (e) of the two complementary experiments performed in 2012 and 2017. The control and the four treatment sets where the colonies were placed were composed of three replicates plus one large buffer tank, which was supplied with filtered seawater. These buffer tanks pumped seawater continuously to the experimental tanks and were used to control their temperature. (*)In the first experiment (2012), the treatments lasted 21, 21, 10 and 8 days respectively. In the second experiment (2017), the latter two treatments (28 and 29°C) were extended to 21 days to ensure the same exposure time to thermal stress across treatments while increasing the amount of information obtained from the experiment as much as possible. Photos by: Eneko Aspillaga

Two complementary experiments were conducted in June‐July 2012 and 2017 with specimens from two different sets of species collected at depth of 15–20 m in Medes Islands MPA (Spain, NW Mediterranean; see Figure 1). In the first experiment, conducted in 2012, individuals of *Leptopsammia pruvoti,*the apical tips (3–5 cm) of *Alcyonium acaule*colonies and fragments of *Petrosia ficiformis, Dysidea avara*and *Crambe crambe*were sampled. In the second experiment, conducted in 2017, the sampling included medium‐sized colonies containing 10 to 15 polyps from the “stocky” and “slender” morphotypes of *Parazoanthus axinellae,*7 cm apical fragments from *Axinella polypoides,*specimens of *Axinella damicornis,*fragments of *Agelas oroides*and medium‐sized colonies ~3–5 cm in diameter from *Cystodytes dellechiajei*. Overall, five species were tested in 2012 and six in 2017, with sampling effort limited as much as possible to reduce disturbance to the populations while maintaining the robustness of the experimental design. In total, samples from 45–75 healthy adult specimens from each species were collected. Moreover, all samples were chosen to have a similar size between 3 and 7 cm (depending on the species) to be easy to manipulate, comparable and suitable for aquaria experiments.

After collection, the specimens were immediately transported in coolers to the Aquarium Experimental Zone of the Institute of Marine Sciences in Barcelona (ICM‐CSIC) where they were placed into the aquaria and acclimated for one week in an open system with 50 μm sand‐filtered running seawater at a natural temperature (17–18°C). The *Leptopsammia pruvoti, Alcyonium acaule, Crambe crambe, Petrosia ficiformis, Dysidea avara and Agelas oroides*samples were fixed to a plastic net as a surface by thin rubber bands. The *Parazoanthus axinellae, Axinella damicornis and Cystodytes dellechiajei*specimens were fixed to glass plates using coral putty (Coralfix Superfast, Grotech). Finally, the *Axinella polypoides*tips were fixed to experimental plates using stainless steel needles of 0.30 mm in diameter to keep them in a vertical position similar to what they present in the field.

### 2.4 Experimental design and setting

To assess the response of the selected species to thermal stress, four temperature treatments were established at 26, 27, 28 and 29°C for periods that ranged from 8 to 21 days. In the experiment conducted in 2012, the treatments lasted 21, 21, 10 and 8 days, respectively. In 2017, the latter two treatments (28 and 29°C) were extended to 21 days to have the same exposure time to thermal stress across treatments while increasing the amount of information obtained from the experiment as much as possible (see Figure 1b, d). These temperature treatments were selected with the specific goal of determining and comparing the thermotolerance responses and thresholds of the species and after no signs of necrosis were observed in previous trials performed in both years at a lower temperature of 25°C (21 days). Moreover, the chosen treatments represent extreme conditions that have sporadically been observed in the Mediterranean Sea during MMEs and are expected to occur more frequently in the future: short periods (<10 days) with a high mean temperature reaching more than 27°C, and long periods (approximately three weeks) at a warm temperature (≥ 25°C; Bensoussan, Romano, Harmelin, & Garrabou, 2010; Crisci et al., 2011; Galli, Solidoro, & Lovato, 2017).

The thermotolerance experiments involved five aquarium sets that corresponded to one control and four treatment tanks. Each aquarium set was composed of three replicates (3 tanks of 70 L each) where 5–6 species and 3–5 individuals per species were placed, plus one buffer tank (70 L) that was used to control the temperature of the water. The buffer tank was supplied with 50‐μm sand‐filtered Mediterranean seawater (pumped from 15 m depth), and from there, the water was carried directly into the experimental tanks, functioning as an open system (see Figure 1e). In addition, every tank was equipped with submersible pumps to facilitate water circulation as well as individual heaters, temperature controllers and HOBO temperature data loggers (registering temperatures every 10 min) to monitor the temperatures throughout the experiment. During the entire duration of the experiment, the specimens were fed three times per week by combining 3ml of a liquid mixture of particles between 10 to 450 μM in size (Bentos Nutrition Marine Active Suplement, Maim, Vic, Spain) in each tank on days 2 and 6 and a tablet of frozen cyclops (Ocean nutrition, Antwerp, Belgium) on day 4.

The thermotolerance experiments encompassed one acclimatization week at 17–18°C for all the aquaria. After this period, the temperature of the control set remained constant (17–18°C), while it was increased in the treatment sets for one week at daily acclimation rates of 1.14, 1.29, 1.43 and 1.57°C (at a common rate of 0.5°C per hour in every treatment within the same day) until reaching 26, 27, 28 or 29°C, respectively. Then, the temperatures were kept constant for a maximum of 21 days (Figure 1b, d). We decided to acclimate the species exposed to different temperature treatments during the same period of one week rather than at the same rate to keep them exposed to experimental conditions the exact same number of days throughout the experiments and given that previous studies dealing with other related co‐occurring species from coralligenous assemblages showed no effect of different acclimation rates in the resulting upper thermal limits of the studied species (Crisci et al., 2017; Torrents et al., 2008).

### 2.5 Experiment response variable

As the response variable, we measured the percentage of necrotic tissue in each specimen, which was visually monitored on a daily basis. This variable has been widely used for coralligenous assemblages both in field mortality assessments and laboratory experiments as a proxy of partial and/or total mortality following disturbance (Cebrian, Uriz, Garrabou, & Ballesteros, 2011; Cerrano et al., 2000; Crisci et al., 2017; Garrabou et al., 2009; Kersting, Bensoussan, & Linares, 2013; Pagès‐Escolà et al., 2018). Following these similar previous studies, we considered necrotic tissue to be all areas with evident signs of irreversible damage, which may include the following: the loss of tissue, denudation/exposure of the skeleton, the loss of coloration derived from the total or partial loss of living tissue and/or the colonization by saprophytic microorganisms (see Supporting information Figure S2). The samples displaying no signs of damage were scored as 0% necrotic, while those exhibiting total tissue necroses were scored as 100%. From these data, we obtained the following indicators for each species: the percentage of affected specimens (those presenting a tissue necrosis percentage >0%) per day, the mean percentage of tissue necrosis per day (calculated as the average percentage of tissue necrosis of all the specimens from the same species pooled together in a given day), the period (in days) until the first signs of necrosis were detected, and finally, the period (in days) until 50% of the specimens showed necrosis. We used these different metrics because they describe both the timing and magnitude of the necrosis of species exposed to thermal stress. Eventually, for the ascidian *Cystodytes dellechiajei,*the level of tissue necrosis at 28 and 29°C could not be accurately assessed due to the incipient process of fission that some colonies suffered throughout these two treatments, which usually started with tissue loss in the part where the colony would end up fragmenting (Supporting information Figure S2j). Previous studies have shown that in the field, fissions of *Cystodytes dellechiajei*colonies are not rare and do not seem to occur following any clear temporal pattern throughout the year. However, they usually precede the death of colonies (López‐Legentil, Ruchty, Domenech, & Turon, 2005). In our experiments, fissions were only observed at 28 and 29°C, indicating that they might have been triggered by thermal stress. However, as this was not explicitly tested and the observed loss of tissue seemed to be related in many colonies to these fissions instead of to a direct lethal effect of warming, the onset of the necrosis was unclear, and this species was excluded from the analysis at these two temperatures.

### 2.6 Statistical analysis

To further characterize the differences in the thermal responses, we used the Kaplan–Meier product limit method (Kaplan & Meier, 1958) and the log‐rank test (Mantel, 1966), which are commonly used in exposure‐dose survival assays across different scientific disciplines as a method for constructing and statistically comparing time to event data curves (De Clercq, 2010; Dickel, Münch, Amdam, Mappes, & Freitak, 2018; Govaert et al., 2012). In the analysis, we focused on the probability of each species of not suffering necrosis through time when exposed to different temperature treatments, which allowed us to determine the upper thermal limits of each species (defined here as the first temperature at which a given species presents a significantly lower probability of remaining healthy (without necrosis) throughout the experiment compared to the control conditions). We could then classify them according to these thermal limits into three different groups: highly resistant (thermal limit ≥28°C), intermediately resistant (thermal limit = 27°C), and minimally resistant or sensitive (thermal limit = 26°C). Furthermore, when significant differences between the control and any of the treatments were obtained, pairwise log‐rank comparison tests were performed to further explore the differences between the treatments (Supporting information Table S3). Then, the same procedure was repeated to explore the differences between the different species and phyla exposed to the same temperature treatments. In this case, only the 10 and 8 day periods were considered when comparing the species or phyla at 28 and 29°C, as this was the duration of the experiment for the species tested in 2012 at these two temperatures. Statistical analyses were not performed regarding the growth form given the low number of species in each group. All the statistical analyses and graphics were produced using R version 3.1.2 (R Core Developer Team, 2014).

## 3 RESULTS

### 3.1 Interspecific responses to thermal stress

**Figure 2:**
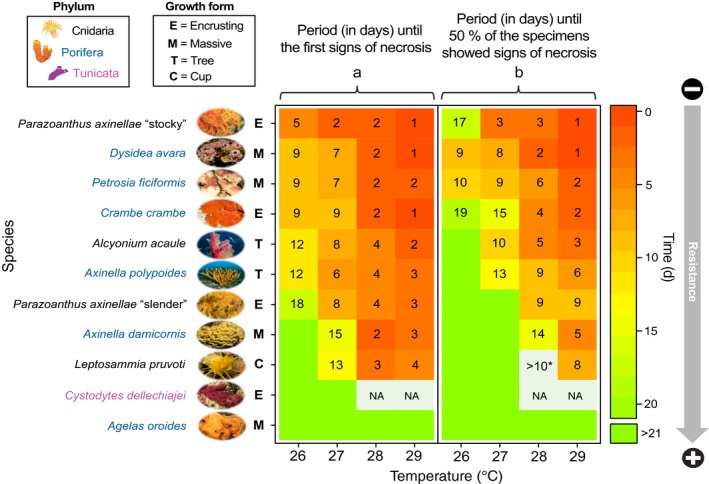
Period (in days) until the first signs of necrosis (a) and until 50% of the specimens showed signs of necrosis (b) for every species and every temperature tested (26, 27, 28 and 29°C). The species have been ordered from least to most resistant, with red colored cells representing those with a higher sensitivity to warming and green colored cells representing those with a higher resistance. The species scientific names appear in blue, black or pink if they are cnidarians, poriferans or tunicates, respectively, and are followed by a letter code that indicates their typical growth form (C = cup; E = encrusting; M = massive and T = tree). NA; data not available. *>10; For *Leptopsammia pruvoti*, (tested in 2012), the experiment at 28°C lasted 10 days and ended before 50% of individuals were affected

**Figure 3:**
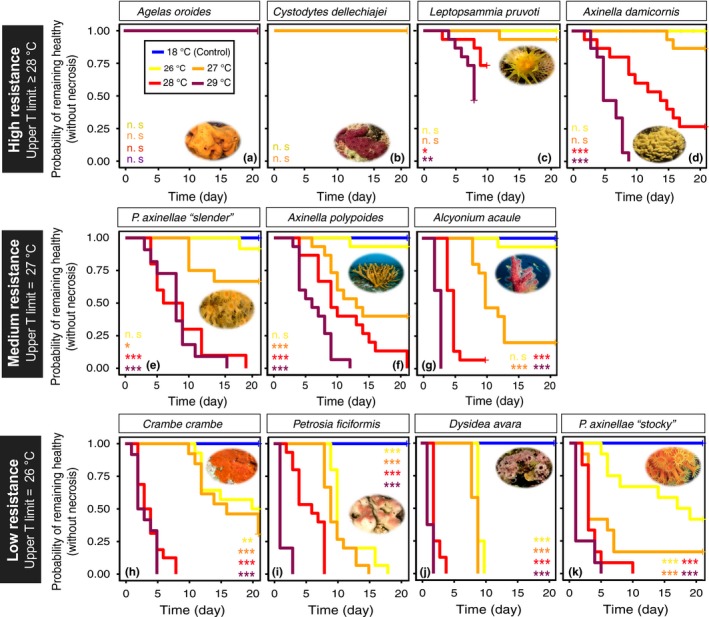
Species‐specific Kaplan–Meier estimated survival curves (referred to as the probability of remaining necrosis‐free through time) for each species (a–k) exposed to the different experimental conditions. For *C. dellechiajei*, only the 18, 26, and 27°C treatments are represented. Furthermore, regarding the species tested in 2012 at 28 and 29°C (whose treatments only lasted 10 and 8 days, respectively; c, g, h, i, j), only these two periods were considered for statistical comparisons at these temperatures. The significance levels of the differences between the control (18°C) and each of the treatments (26, 27, 28 and 29°C) are represented for each species, as follows: ***p‐value <0.001, **p‐value <0.01, *p‐value <0.05 and ns: not significant. Finally, the species have been classified in different groups of resistance according to their upper *T* (thermal) limit (considered here as the first temperature at which a given species presents a lower probability of remaining healthy throughout the experiment compared to the control conditions)

**Figure 4:**
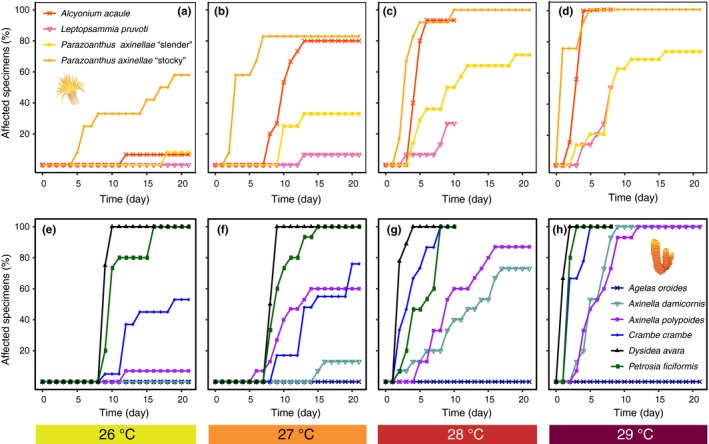
Temporal development of the percentage of affected specimens in the studied cnidarian (above: a, b, c, and d) and porifera (below: e, f, g and h) species for every temperature treatment (26°C, 27°C, 28°C and 29°C) throughout the 21 days of exposure to thermal stress. Each species is represented by a different colored line, and the temperature treatments are represented by different colored boxes. Since all of the control specimens remained healthy without signs of necrotic tissue throughout the experimental period, the control is not represented. *For species tested in 2012, the two warmest treatments (28 and 29°C) lasted only 10 and 8 days, respectively

**Figure 5:**
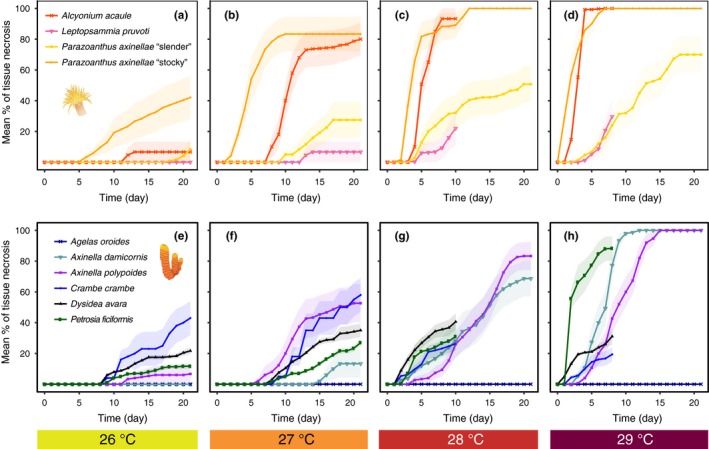
Temporal development of the extent of tissue necrosis (mean ± *SE*) in the studied cnidarian (above: a, b, c and d) and porifera (below: e, f, g and h) species in every temperature treatment (26°C, 27°C, 28°C and 29°C) throughout the 21 days of exposure to thermal stress. Each species is represented by a different colored line, and temperature treatments are represented by different colored boxes. Since all the control specimens remained healthy without signs of necrotic tissue throughout the experimental period, the control is not represented. *For species tested in 2012, the two warmest treatments (28 and 29°C) lasted only 10 and 8 days, respectively

The coralligenous species tested in this study showed differences in both the magnitude and timing of necrosis when submitted to thermal stress, indicating contrasting responses to warming (Figures 2, 4 and 5) and different upper thermal limits (Figure 3). Indeed, significant differences in the probability of remaining healthy through time between the species were found at every temperature treatment (*p* < 0.001; Supporting information Figure S3).

The most resistant species to warming was the sponge *Agelas oroides*, which did not show any kind of necrotic tissue throughout any of the treatments (Figures 2 and 3). Conversely, the most sensitive species was the stocky morphotype of the cnidarian *Parazoanthus axinellae,*which showed the first signs of necrosis after only 5 days at 26°C and after only 1–2 days at any of the higher temperatures (Figure 2a). Between these extremes, a whole range of different responses to thermal stress was found, including highly resistant species (upper thermal limit ≥28°C), species with an intermediate response (upper thermal limit = 27°C) and sensitive species (upper thermal limit = 26°C) (Figure 3).

#### 3.1.1 Species‐specific response patterns

Among the poriferans, *Axinella damicornis* and especially *Agelas oroides* were highly resistant (Figures 2 and 3a, d). The former was not affected when exposed to 26°C (21 days) and needed two weeks to show the first signs of necrosis at 27°C (Figure 2a). Moreover, at the end of this treatment, it displayed low mean levels of necrosis (13.3% ± 9.1; mean ± *SE*; Figure 5f). However, in contrast to *Agelas oroides,* many of the *Axinella damicornis* specimens were affected at 28 and 29°C (Figure 4g, h). With a lower degree of resistance, the sponge *Axinella polypoides*presented an intermediate response, mostly resisting 21 days at 26°C but showing notable percentages of affected specimens and mean tissue necrosis at any higher temperature (Figure 3f and 4e‐h). Eventually, the sponges *Crambe crambe, Dysidea avara,*and *Petrosia ficiformis*displayed a sensitive response to thermal stress (Figure 3h–j). These three sponges showed signs of necrosis from 7 to 9 days at 26 and 27°C and from 1 to 2 days at any higher temperature (Figure 2). Most importantly, even in the lowest‐temperature treatment, every *Dysidea avara*and *Petrosia ficiformis* specimen suffered necrosis (Figure 4e). On its part, although the percentage of affected specimens at 26°C was lower in *Crambe crambe* (Figure 4e)*,* this sponge was the one with the highest mean percentage of necrotic tissue at this temperature (43.2% ± 11.1; mean ± *SE*; Figure 5e).

Among the cnidarians, the most resistant species was *Leptopsammia pruvoti,*which, similar to the sponge *Axinella damicornis,*presented 28°C as its upper thermal limit (Figure 3c). Moreover, even after 8 days of exposure to the highest temperature treatment of 29°C, the mean percentage of tissue necrosis remained relatively low (29.7% ± 10.80; mean ± *SE*; Figure 5d). With a lower degree of resistance, the “slender morphotype” of *Parazoanthus axinellae* and *Alcyonium acaule*presented an intermediate tolerance (Figure 3e, g). Although these two species withstood 21 days at 26°C with very few specimens affected, both showed large percentages of affected specimens and mean tissue necrosis at any higher temperature (Figure 4a–d and 5a–d). In fact, *Alcyonium acaule* followed a similar pattern to the sensitive stocky morphotype of *Parazoanthus axinellae*at 27, 28 and 29°C*,*suffering substantial injury (>80% of tissue death) in most of its colonies relatively quickly after the first signs of necrosis appeared (Figure 5b–d).

Finally, although the development of necrosis at 28 and 29°C could not be accurately assessed for the ascidian *Cystodytes dellechiajei,*this species withstood 26 and 27°C (21 days) without showing signs of necrosis and was therefore among the highly resistant species (Figure 3b).

### 3.2 Intraspecific responses to thermal stress (the case of *Parazoanthus axinellae)*

Interestingly, the two morphotypes of *Parazoanthus axinellae* presented contrasting responses to warming. While the “slender” morphotype was barely affected when placed at 26°C (first signs after 18 days) and did not show any signs of necrosis until 8 days at 27°C (26% mean necrosis at the end of the experiment), the “stocky” morphotype was the most sensitive species of all, showing signs of necrosis after only 5 days at 26°C and after 1–2 days at a higher temperature, which was followed by a rapid increase in the tissue necrosis in many of its individuals (up to 100%; Figures 2, 4a–d and 5a–d). In addition, the probability of suffering necrosis with time significantly differed for these two morphotypes in every temperature treatment (*p* < 0.001; Supporting information Table S4).

### 3.3 Response patterns according to phyla and morphological groups

Despite the great differences observed between the species, we did not observe clear patterns indicating a higher thermotolerance of poriferans versus cnidarians or vice versa. In fact, the probability of displaying necrotic specimens for these two phyla only differed at 26°C (*p* < 0.001), while no significant differences were found at 27, 28 or 29°C (*p* = 0.71; *p* = 0.63 and *p* = 0.65, respectively; Supporting information Figure S4). Most importantly, when considering the percentage of affected specimens or the trends in mean percentage of tissue necrosis over time in every treatment, most of the differences were found within each group, with both poriferans and cnidarians showing resistant and sensitive species (Figures 4 and 5). Eventually, although statistical analyses could not be performed regarding the growth form given the low number of species in each group, the different upper thermal limits shown by the species with an equivalent morphology suggests that contrasting responses to warming also occur between species with similar structural roles (Figures 2 and 3).

## 4 DISCUSSION

Forecasting temperature effects on ecological communities require a deep understanding of how temperature may influence the physiology of their different members. Thus, as oceans keep warming, community‐wide thermal sensitivity studies are becoming a powerful tool for reducing the uncertainty about the future composition, structure and functionality of marine communities facing climate change (Beveridge, Petchey, & Humphries, 2010; Fey & Cottingham, 2012; Iles, 2014; Savva et al., 2018; Stuart‐Smith, Edgar, Barrett, Kininmonth, & Bates, 2015). In this study, we explored the ranges of thermal sensitivity among structurally, functionally and taxonomically different components of Mediterranean coralligenous assemblages, showing contrasting responses to warming and suggesting the presence of potential “winners” and “losers” in the face of climate change.

### 4.1 Contrasting responses from experimental studies

In our study, the experimental responses to thermal stress ranged from completely resistant species that did not suffer necrosis in any of the treatments (>21 days at 29°C) to sensitive species, which suffered necrosis after short‐term exposure (5–9 days) to the lowest‐temperature treatment of 26°C. In between these extremes, different levels of tolerance were found, including highly resistant species, for which 28°C represented the upper thermal limit that significantly reduced their probability of not suffering mortality, and intermediately tolerant species, which despite being affected at 26°C, did not suffer generalized necrosis until 1 week of exposure to 27°C. Bearing in mind that summer heat waves capable of sustaining temperatures between 26°C and 29°C for several days might become increasingly frequent during the next decades in diverse NW Mediterranean locations (Galli et al., 2017), our results suggest potential differences in climate change vulnerability among co‐occurring species dwelling in coralligenous assemblages. Indeed, differences between the members of the community were observed in our experiments to the extent that even the two morphotypes of *Parazoanthus axinellae*presented contrasting thermal responses. Regardless of the possible mechanisms behind these differences, which in the case of *Parazoanthus axinellae*could include the presence of highly bioactive secondary metabolites only in the “slender” morphotype as chemical defences induced for coping with environmental changes (Cachet et al., 2015; Reverter et al., 2016), our results represent a good example of the diversity of responses to warming found among structurally, functionally and taxonomically related organisms dwelling in coralligenous outcrops. Previous studies dealing with habitat‐forming emblematic species from these assemblages, such as the red gorgonian *Paramuricea clavata* (Risso, 1826), the white gorgonian *Eunicella singularis* (Esper, 1791), the red coral *Corallium rubrum* (Linnaeus, 1758) or the bryozoans *Myriapora truncata*(Pallas, 1766) and *Pentapora fascialis*(Pallas, 1766), have already pointed to such diversity (Crisci et al., 2017; Linares et al., 2013; Pagès‐Escolà et al., 2018; Torrents et al., 2008). However, the low number of studied species impeded the assessment of whether the response diversity was limited or widespread at the community level. Our results reinforce the latter possibility and suggest that regardless of their phyla and/or structural role, some species from these habitats could be living closer to their thermal limits than others and therefore might be more vulnerable under future warming scenarios.

### 4.2 Linking experimental and observational studies: evidence from MMEs

The high variability of thermal responses observed in our study contributes to explaining why some coralligenous species were more affected than others in previous MMEs linked to warming (Cerrano et al., 2000; Garrabou et al., 2009; Linares et al., 2017; Perez et al., 2000). Moreover, the high (or low) resistance shown by the species in our thermal experiments was, in most cases, concomitant with the high (or low) vulnerability shown by these species in the past during these warming events (Table 1). For instance, species such as *Axinella damicornis* or *Leptopsammia pruvoti* that were highly resistant in our aquaria have never been reported as affected during previous warming‐induced MMEs that occurred in the NW Mediterranean Sea. In contrast, other species, such as *Petrosia ficiformis, Crambe crambe, Alcyonium acaule*or *Parazoanthus axinellae,*which have been greatly impacted during previous warming events (Cerrano et al., 2000; Cerrano, Magnino, Sarà, Bavestrello, & Gaino, 2001; Cerrano, Totti, Sponga, & Bavestrello, 2006; Garrabou et al., 2009; Linares et al., 2017; Parravicini et al., 2010; Perez et al., 2000), showed a higher vulnerability to thermal stress in our experiments. Thus, as we hypothesized, species‐specific thermal tolerances seem to play an important role in shaping divergent vulnerabilities in coralligenous species exposed to marine heat waves. Nonetheless, the unexpected differences in the responses among the aquaria thermotolerance experiments and observations in the field are also notable in some cases. In our experiment, *Agelas oroides* presented the highest resistance to thermal stress (> 21 days at 29°C) despite having sporadically been impacted during previous warming‐induced MMEs that were triggered at lower temperatures (Garrabou et al., 2009). Conversely, *Dysidea avara*was one of the most sensitive species in our experiment, while to our knowledge, no records of mass mortality linked to marine heat waves exist for this species in the NW Mediterranean. Such paradoxes have been noted in previous studies on mass mortality and bleaching events both in tropical and temperate species and have been attributed to the multifactorial nature of these events. A clear example of this is the Mediterranean coral *Cladocora caespitosa*(Ehrenberg, 1834), which, despite suffering recurrent warming‐induced mass mortalities in the field, showed resistance in single factor (temperature) experiments performed in aquaria while being impacted when exposed to additional factors such as the presence of invasive species (Kersting et al., 2015). Other factors that have been highlighted include food availability, pathogens, genetic differences or different physiologic processes (Arizmendi‐Mejía et al., 2015; Cebrian et al., 2011; Crisci et al., 2017; Linares et al., 2013; Pivotto et al., 2015). Therefore, bearing in mind the complex network of interacting factors that may ultimately determine vulnerability to warming in the field, determining the absolute thermal limits before which mortality of a given species should not be expected remains challenging. Our goal was, instead, to provide a ranking of thermal sensitivities among key components of coralligenous assemblages that could serve as a valuable baseline for better understanding the capacity of response and the trajectories of these species over broad temporal and spatial scales.

**Table 1:** The thermal tolerances of the studied species confronted to observations from MMEs linked to warming in the NW Mediterranean Sea and reported in the scientific literature (1979–2017)

Species	Phylum	Growth form	Upper thermal limit in aquaria (°C)	Resistance in aquaria	Degree of damage in MMEs (NW Mediterranean)
*Agelas oroides*	Poriferan	Massive	>29°C (21 days)	High	*
*Cystodytes dellechiajei*	Tunicate	Encrusting	>27°C (21 days)	High	No reported damage
*Leptopsammia pruvoti*	Cnidarian	Cup	28°C	High	No reported damage
*Axinella damicornis*	Poriferan	Massive	28°C	High	No reported damage
*Axinella polypoides*	Poriferan	Tree	27°C	Medium	No reported damage
*Alcyonium acaule*	Cnidarian	Tree	27°C	Medium	*
*Crambe crambe*	Poriferan	Encrusting	26°C	Low	***
*Petrosia ficiformis*	Poriferan	Massive	26°C	Low	***
*Dysidea avara*	Poriferan	Massive	26°C	Low	No reported damage
*Parazoanthus axinellae*	Cnidarian	Encrusting	27°C “slender”	Medium	***
			26°C “stocky”	Low	

The species have been ordered from the most to the least resistant according to their upper thermal limits obtained in aquaria (considered here as the first temperature significantly reducing their probability of remaining healthy without necrosis throughout the experimental period). No reported damage indicates that a given species has not been reported as being affected in any MME, whereas (*) refers to species that have been reported as being affected only in one MME and (***) refers to species that have been affected in multiple MMEs (>5 years and/ or locations). See Supporting information & Table S2 for further detail and references.

### 4.3 Response diversity in coralligenous assemblages facing climate change: consequences for ecosystem structure and functioning

According to the insurance hypothesis of biodiversity (Gonzalez & Loreau, 2009; Yachi & Loreau, 1999), a high “response diversity” among functionally redundant organisms is essential for buffering the effects of environmental changes and ensuring the ecosystem functionality that prevents regime shifts (Mori et al., 2013). In the highly diverse coralligenous outcrops, the diversity of responses shown by the different studied species (including different phyla and different structural roles) suggests that rather than suffering dramatic shifts in response to climate change, many of these assemblages could be susceptible of changing their configuration in the future to less diverse but functionally similar systems, where thermally sensitive species might be replaced by more resistant species. However, the final outcomes will not depend only on species‐specific thermal tolerances. The “winners” and “losers” will also depend on the great variety of specific life histories and functional traits they present, and how these traits favor, or impair their success in a changing sea (Darling, Alvarez‐Filip, Oliver, McClanahan, & Côté, 2012; Hughes et al., 2018; Madin et al., 2016; Van Woesik, Sakai, Ganase, & Loya, 2011). For instance, populations of species that present faster growth and dispersal or higher reproduction may recover faster after disturbances and/or adapt more easily to rapid environmental changes over generations than populations of species with traits related to a close adaptation to their current environment, extremely slow dynamics or a low dispersal capacity (McKinney & Lockwood, 1999). Likewise, changes in ecological interactions may also determine the final result, as species might be ultimately favored or disfavored by the tolerant or sensitive response of others with which they are ecologically connected (Walther, 2010). For instance, the loss of key habitat‐forming species (such as some sensitive gorgonians) could potentially trigger cascading effects on the community that could result in an overall reduction in structural complexity and resilience (Ponti et al., 2014). Similarly, a decline in some massive slow‐growing sponges, such *Petrosia ficiformis*, could exert a major influence on the overall ecosystem functioning and stability (Bell, 2008). Therefore, whether the eventual “winners” will be able to replace the “losers” in such a way that the complexity and functioning of the coralligenous assemblages are maintained in the future warmed Mediterranean Sea remains to be seen. The contrasting responses to warming among the different components of these assemblages unravelled in this study indicate some promising capacity to buffer future warming effects. However, steering coralligenous outcrops in a way that their functionality is safeguarded in the face of climate change will be challenging and will necessarily depend upon an extensive understanding of the interplay between the functional and life history traits of coralligenous key species, their ecological interactions and their species‐specific vulnerabilities to climatic disturbances.

## CONFLICT OF INTEREST

On behalf of all authors, the corresponding author states that there is no conflict of interest.

## AUTHOR'S CONTRIBUTIONS

D.G.G, C.L, E.C and J.G conceived the ideas and designed methodology. D.G.G, C.L, E.C, S.C, I.M.S, M.P.E and J.G collected samples in the field. D.G.G, M.F.V, S.C, P.L.S and M.P.E performed thermotolerance experiments in aquaria. D.G.G and S.C analyzed the data. D.G.G led the writing of the manuscript, and all authors contributed critically to the drafts and gave final approval for publication.

## Data Availability

Data can be accessed on the Dryad repository: (https://doi.org/10.5061/dryad.6d4p06g).
