# Supplementary material for: Response diversity in Mediterranean coralligenous assemblages facing climate change: Insights from a multispecific thermotolerance experiment
Source: Ecol Evol. 2019 Mar 12;9(7):4168–80. doi: 10.1002/ece3.5045 (PMC6468064; doi:10.1002/ece3.5045)
Supplement: Supplementary file 1 [file ECE3-9-4168-s001.docx]

**SUPPLEMENTARY MATERIAL**

**Response diversity in Mediterranean coralligenous assemblages facing climate change: insights from a multi-specific thermotolerance experiment**

Daniel Gómez-Gras^1*^, Cristina Linares^2^, Sonia de Caralt^3,4^, Emma Cebrian^3,4^, Maša Frleta-Valić^1^, Ignasi Montero-Serra^2^, Marta Pagès-Escolà^2^, Paula López-Sendino^1^ and Joaquim Garrabou^1^

^1^ Departament de Biologia Marina, Institut de Ciències del Mar (CSIC), Barcelona, Spain

^2^ Departament de Biologia Evolutiva, Ecologia i Ciències Ambientals, Institut de Recerca de la Biodiversitat (IRBIO), Universitat de Barcelona, Barcelona, Spain.

^3^ Centre d' Estudis Avançats de Blanes (CSIC), Blanes, Spain

^4^ GR MAR, Institut d’Ecologia Aquàtica, Facultat de Ciències, Universitat de Girona, Girona, Spain.

*Corresponding author: danielgomez@icm.csic.es

**Supplementary methods (I):** Additional information on the model species

**Figure S1.** Model species

**Figure S2.** Photographs of of healthy specimens vs affected specimens

**Figure S3.** Kaplan-Meier estimated survival curves for all species pooled together exposed to every temperature treatment

**Figure S4.** Kaplan-Meier estimated survival curves for cnidarians and poriferans exposed to every temperature treatment

**Table S1.** Descriptions of the growth forms of the studied species

**Table S2.** Table reporting all documented MMEs of the species tested in this study occurred across the NW Mediterranean Sea from 1983 to 2017

**Table S3.** Post hoc pairwise comparison results of the log-rank test performed to further characterize the differences between the treatments for each species

**Table S4.** Post hoc pairwise comparison results of the log-rank test performed to further characterize the differences between the species

**Supplementary methods S1. Additional information on the model species**

*Leptopsammia pruvoti* (Lacaze-Duthiers, 1897) is a solitary azooxanthellate (cup-shaped) scleractinian coral with a geographical distribution along the Mediterranean Sea and Atlantic coast (from Portugal to southern England) and a depth range from the surface to 70 m (Zibrowius, 1980). *Alcyonium acaule* (Marion, 1878) is a red colonial alcyonacean with a massive treelike growth form inhabiting the north-western (NW) Mediterranean Sea within a depth range from 10 to 45 m (Gili, Garcia & Colomer, 1984). The so-called yellow cluster anemone, *Parazoanthus axinellae* (Schmidt, 1862)*,* is a zoanthid that can be found on the southern Atlantic coasts and in the Mediterranean Sea from 1 m depth to depths greater than 100 m (Gili, Garcia & Colomer, 1984; Previati et al., 2010). Two different and easily distinguished morphotypes of this species were used in this study; the rather yellow and thin “slender” morphotype, which mostly lives in rocky substrates but can also be usually found as an epibiont on demosponges, and the bright orange “stocky” morphotype, which is mainly found in primary substrate where it forms encrusting dense sheets (Cachet et al., 2015). *Crambe crambe* (Schmidt, 1862) is a poecilosclerid red-orange encrusting sponge that is characteristic of the western Mediterranean Sea, but is also present in the eastern Mediterranean and on the Atlantic coast of Portugal and the Canary Islands, and it has a depth range of 1 to 60 m (Uriz, Rosell & Martin, 1992). *Dysidea avara* (Schmidt, 1862) is a violet-coloured, massive and heavily conulated dictyoceratid sponge common to the Mediterranean Sea from depths of 3 to 80 m (Uriz, Rosell & Martin, 1992). *Petrosia ficiformis* (Poiret, 1979) is a red wine coloured haplosclerid sponge with a massive lobated body that is common in the Mediterranean Sea and Atlantic (Azores, Canary Islands, Cape Verde) from 10 to 40 m depths (Maldonado & Riesgo, 2009). *Agelas oroides* (Schmidt, 1864) is a massive, encrusting, variably lobate-digitate sponge that is generally orange and found in the the eastern Atlantic and Mediterranean waters to a depth of 50 m (Ferreti et al., 2009). *Axinella damicornis* (Esper, 1974) is a yellow sponge (verging on orange at the margins) whose branches fuse to form variable stubby finger-like shapes, and that can be commonly found in coralligenous outcrops of the Mediterranean Sea to 30 m depth as well as in the Atlantic and North Sea (Van Soest, 2001). *Axinella polypoides* (Schmidt, 1862) is a brown to orange erect and arborescent sponge that can reach 1 m in size and can be found from 15 to 100 m in the Atlantic and Mediterranean Sea (Uriz, 1986). Finally, *Cystodytes dellechiajei* (Della Valle, 1877) is a cosmopolitan ascidian composed of many zooides found both in tropical and temperate seas as encrusting colonies of less than 10 cm (López-Legentil, Ruchty, Domenech & Turon, 2005).

**Figure S1.** Model species by phyla (and growth form): Cnidarians; a) *Leptopsammia pruvoti* (cup); b) *Alcyonium acaule* (tree); c) *Parazoanthus axinellae* “stocky” (encrusting) and d) *Parazoanthus axinellae* “slender” (encrusting). Tunicates; e) *Cystodytes dellechiajei* (encrusting). Poriferans; f) *Petrosia ficiformis* (massive); g) *Crambe crambe* (encrusting); h) *Agelas oroides* (massive); i) *Dysidea avara* (massive); j) *Axinella damicornis* (massive) and k) *Axinella polypoides* (tree). Photos by: Eneko Aspillaga.

**
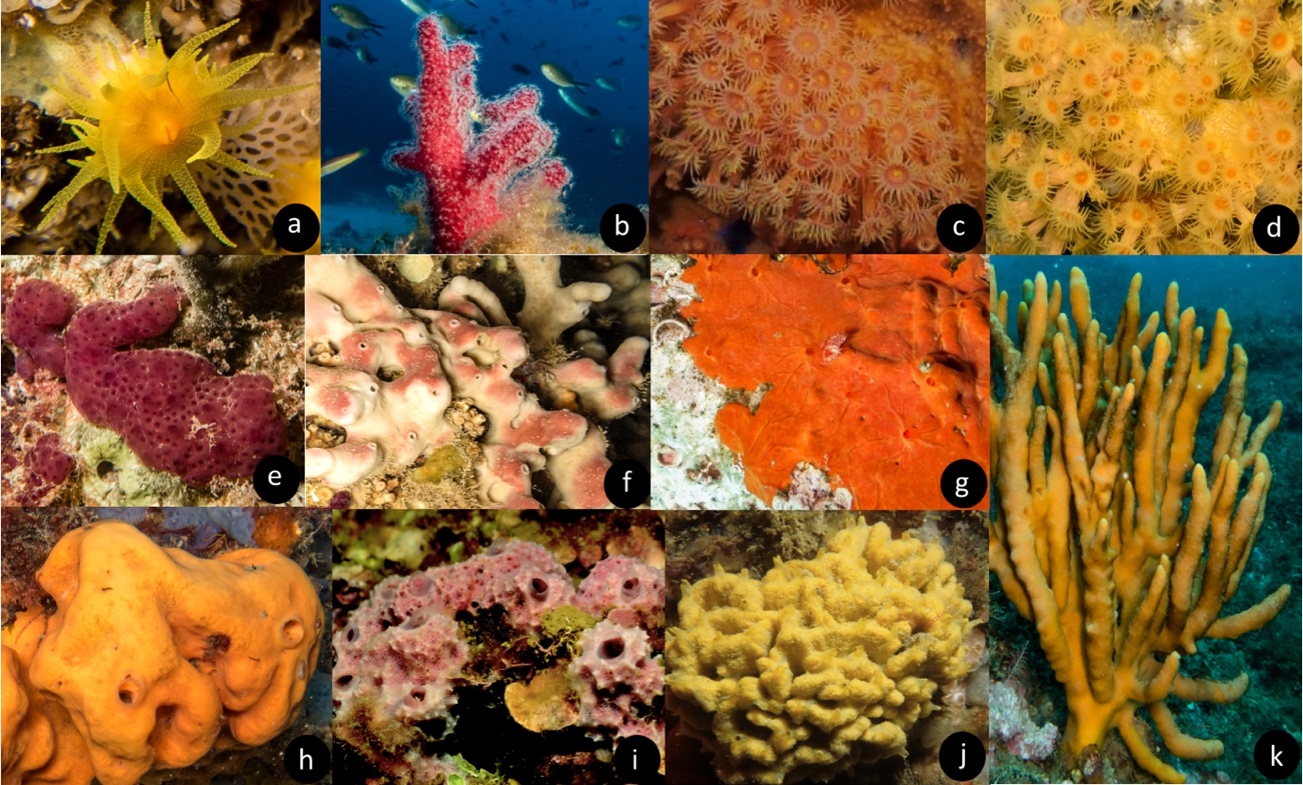
**

**Figure S2.** Photographs of examples of healthy specimens vs affected specimens (percentage of tissue necrosis > 0 %) of each species exposed to thermal stress (a-i). Yellow arrows point to affected tissue. Because no *Agelas oroides* specimens were affected throughout the experiments, this species has not been included in the figure. Regarding *Cystodytes dellechiajei,* the process of fission that some of the colonies experienced at 28 ºC and 29 ºC hindered the assessment of partial/total mortality for this species, which is shown in (j).

**
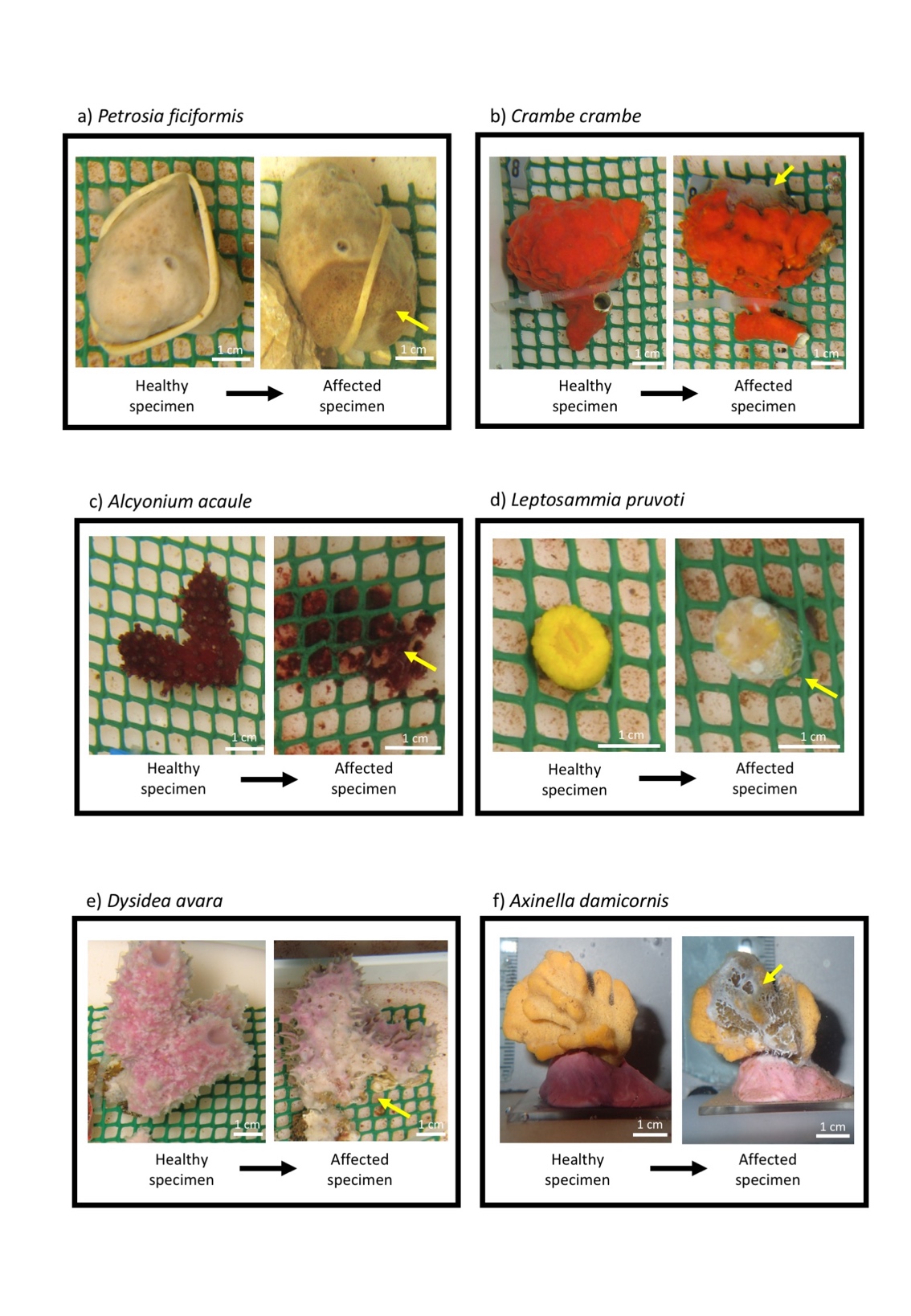
**

**
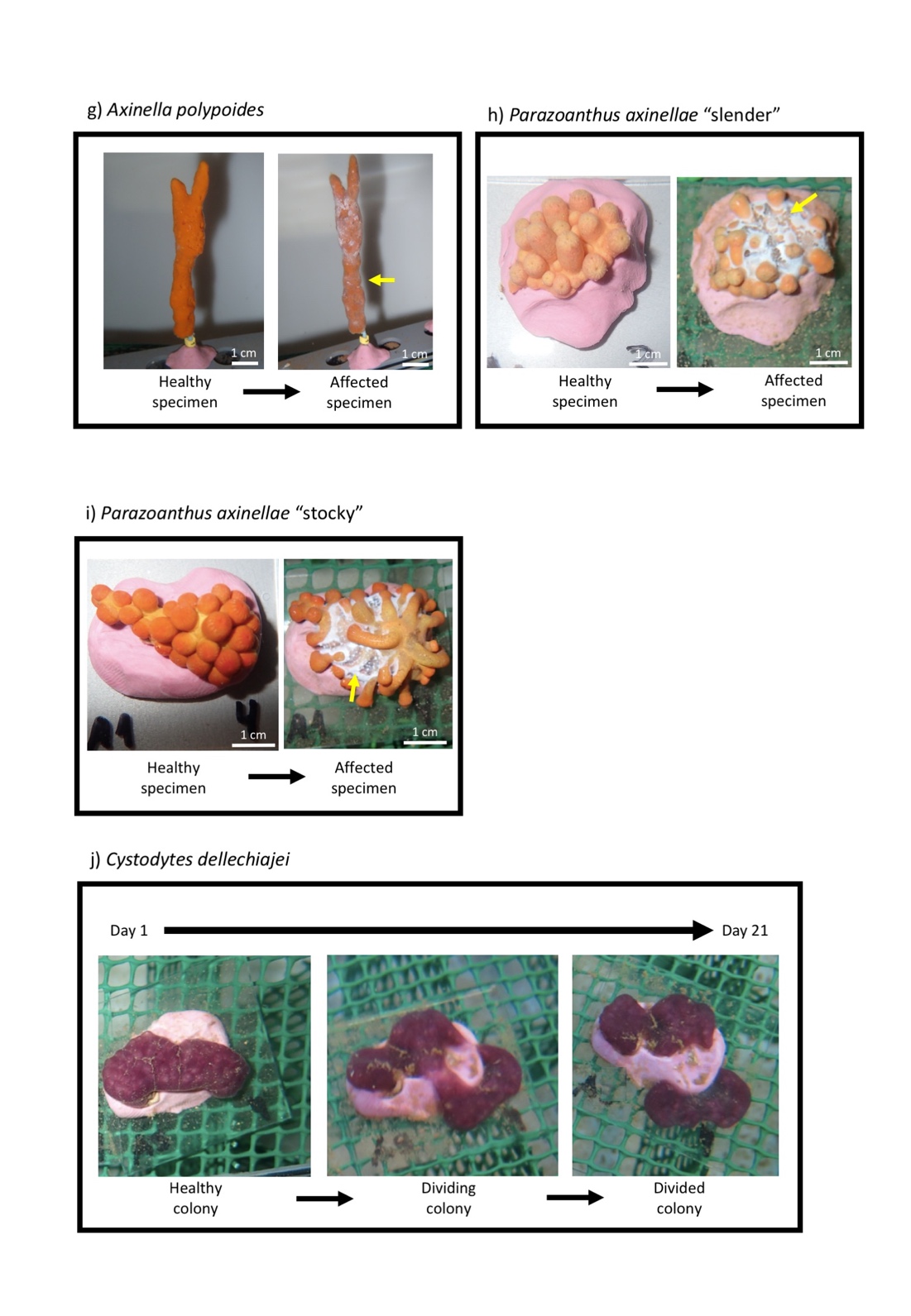
**

**Figure S3.** Kaplan-Meier estimated survival curves (referred to as the probability of remaining necrosis-free through time) for all the species pooled together at each temperature treatment (a-d). Despite the treatments of 28 and 29 ºC lasting 21 days in 2017, only 10 and 8 days, respectively, have been considered in the analysis, as this was the duration of the experiment performed at these temperatures in 2012 and is therefore the period of time in which all of the species can be compared. A p-value < 0.05 (log-rank test) indicates significant differences between species. * *Cystodytes dellechiajei* is only represented at 26 and 27 ºC.

**Figure S4**. Kaplan-Meier estimated survival curves (referred as to the probability of remaining necrosis-free through time) and 95% confidence intervals (shading) for both cnidarians (including four species) and poriferans (including six species) in every temperature treatment (a-d). Despite the treatments of 28 and 29 ºC lasting 21 days in 2017, only 10 and 8 days, respectively, have been considered in the analysis, as this was the duration of the experiment performed at these temperatures in 2012 and therefore is the period of time in which all of the species can be compared. A p-value < 0.05 (log-rank test) indicates significant differences between these two phyla.


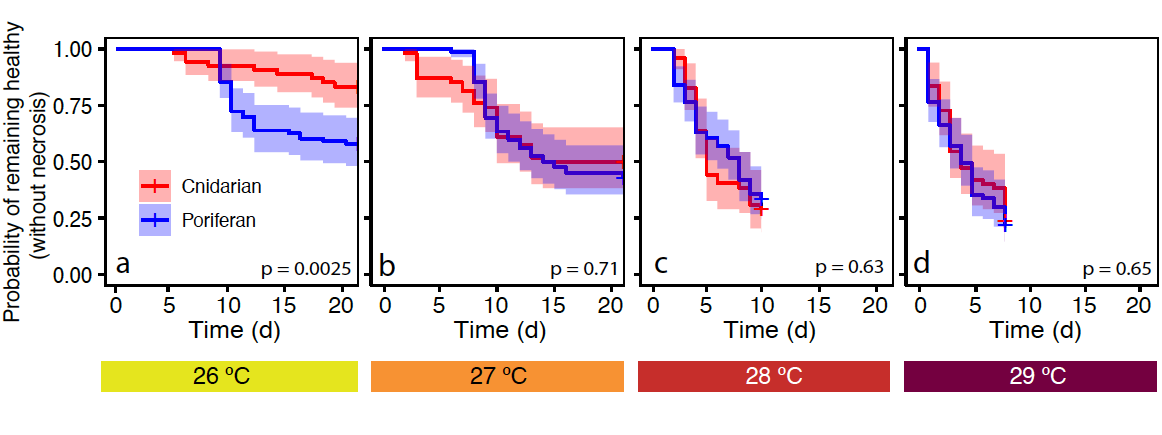


**Table S1. Descriptions of the growth forms of the studied species.** Adapted from Casas-Güell, Teixidó, Garrabou & Cebrian, 2015.

| **Growth forms** | **Description** |
| --- | --- |
| **Cup** | Solitary corals attached to the substratum all along their basal area |
| **Encrusting** | Species growing as two dimensional sheets; more or less completely attached to the substratum |
| **Massive** | Mound species with vertical and lateral growth; normally attached to the substratum along their basal area |
| **Tree** | Erect species more or less branched; usually with a single point of attachment to the substratum |

**Table S2.** Table reporting all the documented MMEs of the species tested in this study occurring across the NW Mediterranean Sea from 1983 to 2017.

| **Year** | **Mediterranean region** | **Sub-basin** | **Location** | **Long.** | **Lat.** | **Start of the event** | **Depth (m)** | **Phyla affected** | **Species affected** | **Identified driver of change (abiotic)** | **Reference** |
| --- | --- | --- | --- | --- | --- | --- | --- | --- | --- | --- | --- |
| 1998 | North-West Mediterranean | Liguo-Provençal | Gallinaria Island | 8,23 | 44,03 | August | 5 | Porifera | *Petrosia ficiformis* | Increase sea water temperature Decrease of salinity | 1 |
| 1999 | North-West Mediterranean | Liguo-Provençal | Bergeggi cave | 8,26 | 44,14 | September | 7 | Porifera | *Petrosia ficiformis* | Increase sea water temperature | 2 |
| 1999 | North-West Mediterranean | Liguo-Provençal | Tino Island | 9,85 | 44,02 | September | 27 | Cnidaria | *Parazoanthus axinellae* | Increase sea water temperature Decrease of salinity Mixed layer depth ~ 40 m | 3 |
| 1999 | North-West Mediterranean | Liguo-Provençal | Mesco Point | 9,64 | 44,1 | September | 20 | Cnidaria | *Parazoanthus axinellae* | Increase sea water temperature Decrease of salinity Mixed layer depth ~ 40 m | 3 |
| 1999 | North-West Mediterranean | Liguo-Provençal | Saint-Tropez | 6,63 | 43,27 | August September | 10 | Porifera | *Crambe crambe* | Increase sea water temperature Mixed layer depth ~ 40 m | 4 |
| 1999 | North-West Mediterranean | Liguo-Provençal | Port-Cros | 6,39 | 42,98 | September | 10 | Porifera | *Crambe crambe* | Increase sea water temperature Mixed layer depth ~ 40 m | 4 |
| 2001 | North-West Mediterranean | Liguo-Provençal | Portofino Promontory | 9,19 | 44,3 | Summer | 10 | Cnidaria | *Parazoanthus axinellae* | Increase sea water temperature | 5 |
| 2001 | North-West Mediterranean | Liguo-Provençal | Gallinaria Island | 8,23 | 44,03 | Summer | 10 | Cnidaria | *Parazoanthus axinellae* | Increase sea water temperature | 5 |
| 2002 | North-West Mediterranean | Liguo-Provençal | Portofino Promontory | 9,19 | 44,3 | Summer | 10 | Cnidaria | *Parazoanthus axinellae* | Increase sea water temperature | 5 |
| 2002 | North-West Mediterranean | Liguo-Provençal | Gallinaria Island | 8,23 | 44,03 | Summer | 10 | Cnidaria | *Parazoanthus axinellae* | Increase sea water temperature | 5 |
| 1999 | North-West Mediterranean | Liguo-Provençal | Bergeggi cave | 8,26 | 44,14 | September | 7 | Porifera | *Petrosia ficiformis* | Increase sea water temperature | 2 |
| 2003 | North-West Mediterranean | Liguo-Provençal | Portofino Promontory | 9,19 | 44,3 | Summer | 10 | Cnidaria | *Parazoanthus axinellae* | Increase sea water temperature | 5 |
| 2003 | North-West Mediterranean | Liguo-Provençal | Gallinaria Island | 8,23 | 44,03 | Summer | 10 | Cnidaria | *Parazoanthus axinellae* | Increase sea water temperature | 5 |
| 2003 | North-West Mediterranean | Liguo-Provençal | Provence coast | 5,35 | 43,29 | August September | 25 | Porifera | *Agelas oroides* | Increase sea water temperature | 6 |
| 2003 | North-West Mediterranean | Liguo-Provençal | Provence coast | 5,35 | 43,29 | August September | 25 | Porifera | *Crambe crambe* | Increase sea water temperature | 6 |
| 2003 | North-West Mediterranean | Liguo-Provençal | Provence coast | 5,35 | 43,29 | August September | 25 | Porifera | *Petrosia ficiformis* | Increase sea water temperature | 6 |
| 2003 | North-West Mediterranean | Liguo-Provençal | Gulf of Genoa | 8,91 | 44,39 | August September | 30 | Cnidaria | *Parazoanthus axinellae* | Increase sea water temperature | 6 |
| 2003 | North-West Mediterranean | Liguo-Provençal | Gulf of Genoa | 8,91 | 44,39 | August September | 30 | Porifera | *Petrosia ficiformis* | Increase sea water temperature | 6 |
| 2003 | North-West Mediterranean | Tyrrhenian | Corsica (Scandola) | 8,55 | 42,35 | August September | 30 | Porifera | *Crambe crambe* | Increase sea water temperature | 6 |
| 2003 | North-West Mediterranean | Tyrrhenian | Sardinia (Bonifacio) | 9,26 | 41,29 | August September | 30 | Porifera | *Crambe crambe* | Increase sea water temperature | 6 |
| 2003 | North-West Mediterranean | Tyrrhenian | Corsica (Scandola) | 8,55 | 42,35 | August September | 30 | Porifera | *Petrosia ficiformis* | Increase sea water temperature | 6 |
| 2003 | North-West Mediterranean | Tyrrhenian | Sardinia  (Bonifacio) | 9,26 | 41,29 | August September | 30 | Porifera | *Petrosia ficiformis* | Increase sea water temperature | 6 |
| 2011 | North-West Mediterranean | Catalan | Cabrera National Park | 2,935 | 39,174 | Summer | 40 | Cnidaria | *Alcyonium acaule* | Increase sea water temperature | 7 |
| 2011 | North-West Mediterranean | Catalan | Cabrera National Park | 2,935 | 39,174 | Summer | 40 | Porifera | *Crambe crambe* | Increase in sea water temperature | 7 |

**Refs: (1) Cerrano, Magnino, Sarà, Bavestrello & Gaino, 2001; (2) Parravicini et al. 2010; (3) Cerrano et al. 2000; (4) Perez et al. 2001; (5) Cerrano, Totti, Sponga & Bavestrello, 2006; (6) Garrabou et al. 2009; (7) Linares et al. 2017.**

**Table S3. Post hoc pairwise comparison results of the log-rank test performed to further characterize the differences between the treatments for each species.** Significance levels: *******: p-value < 0.001, ******: p-value < 0.01**, *:** p-value < 0.05 and **ns**: not significant.

| *A. oroides* | Control (18 ºC) | 26 ºC | 27 ºC | 28 ºC |
| --- | --- | --- | --- | --- |
| 26 ºC | n.s |  |  |  |
| 27 ºC | n.s | n.s |  |  |
| 28 ºC | n.s | n.s | n.s |  |
| 29 ºC | n.s | n.s | n.s | n.s |
|  |  |  |  |  |
| *L. pruvoti* | Control (18 ºC) | 26 ºC | 27 ºC | 28 ºC |
| 26 ºC | n.s |  |  |  |
| 27 ºC | n.s | n.s |  |  |
| 28 ºC | * | * | * |  |
| 29 ºC | ** | ** | ** | * |
|  |  |  |  |  |
| *A. damicornis* | Control (18 ºC) | 26 ºC | 27 ºC | 28 ºC |
| 26 ºC | n.s |  |  |  |
| 27 ºC | n.s | n.s |  |  |
| 28 ºC | *** | *** | *** |  |
| 29 ºC | *** | *** | *** | *** |
|  |  |  |  |  |
| *P. axinellae “slender”* | Control (18 ºC) | 26 ºC | 27 ºC | 28 ºC |
| 26 ºC | n.s |  |  |  |
| 27 ºC | * | n.s |  |  |
| 28 ºC | *** | *** | *** |  |
| 29 ºC | *** | *** | *** | * |
|  |  |  |  |  |
| *A. polypoides* | Control (18 ºC) | 26 ºC | 27 ºC | 28 ºC |
| 26 ºC | n.s |  |  |  |
| 27 ºC | *** | ** |  |  |
| 28 ºC | *** | *** | * |  |
| 29 ºC | *** | *** | *** | ** |
|  |  |  |  |  |
| *A. acaule* | Control (18 ºC) | 26 ºC | 27 ºC | 28 ºC |
| 26 ºC | n.s |  |  |  |
| 27 ºC | *** | *** |  |  |
| 28 ºC | *** | *** | *** |  |
| 29 ºC | *** | *** | *** | ** |
|  |  |  |  |  |
| *C. crambe* | Control (18 ºC) | 26 ºC | 27 ºC | 28 ºC |
| 26 ºC | ** |  |  |  |
| 27 ºC | *** | n.s |  |  |
| 28 ºC | *** | *** | *** |  |
| 29 ºC | *** | *** | *** | n.s |
|  |  |  |  |  |
| *P. ficiformis* | Control (18 ºC) | 26 ºC | 27 ºC | 28 ºC |
| 26 ºC | *** |  |  |  |
| 27 ºC | *** | * |  |  |
| 28 ºC | *** | *** | *** |  |
| 29 ºC | *** | *** | *** | *** |
|  |  |  |  |  |
| *D. avara* | Control (18 ºC) | 26 ºC | 27 ºC | 28 ºC |
| 26 ºC | *** |  |  |  |
| 27 ºC | *** | n.s |  |  |
| 28 ºC | *** | *** | *** |  |
| 29 ºC | *** | *** | *** | n.s |
|  |  |  |  |  |
| *P. axinellae “stocky”* | Control (18 ºC) | 26 ºC | 27 ºC | 28 ºC |
| 26 ºC | ** |  |  |  |
| 27 ºC | *** | *** |  |  |
| 28 ºC | *** | *** | ** |  |
| 29 ºC | *** | *** | ** | * |
|  |  |  |  |  |
| *C. dellechiajei* | Control (18 ºC) | 26 ºC |  |  |
| 26 ºC | n.s |  |  |  |
| 27 ºC | n.s | n.s |  |  |
|  |  |  |  |  |

**Table S4. Post hoc pairwise comparison results of the log-rank test performed to further characterize the differences between the species exposed to the different treatments.** Significance levels: *******: p-value < 0.001, ******: p-value < 0.01**, *:** p-value < 0.05 and **ns**: not significant.

| **26 ºC** | *Agelas oroides* | *Alcyonium acaule* | *Axinella damicornis* | *Axinella polypoides* | *Crambe crambe* | *Dysidea avara* | *Leptopsammia*  *pruvoti* | *Parazoanthus axinellae “slender”* | *Parazoanthus axinellae “stocky”* |
| --- | --- | --- | --- | --- | --- | --- | --- | --- | --- |
| *A. acaule* | n.s |  |  |  |  |  |  |  |  |
| *A. damicornis* | n.s | n.s |  |  |  |  |  |  |  |
| *A. polypoides* | n.s | n.s | n.s |  |  |  |  |  |  |
| *C. crambe* | ** | * | ** | * |  |  |  |  |  |
| *D. avara* | *** | *** | *** | *** | *** |  |  |  |  |
| *L. pruvoti* | n.s | n.s | n.s | n.s | ** | *** |  |  |  |
| *P. axinellae “slender”* | n.s | n.s | n.s | n.s | * | *** | n.s |  |  |
| *P. axinellae “stocky”* | ** | ** | ** | ** | n.s | * | ** | * |  |
| *P. ficiformis* | *** | *** | *** | *** | *** | ** | *** | *** | * |

| **27 ºC** | *Agelas oroides* | *Alcyonium acaule* | *Axinella damicornis* | *Axinella polypoides* | *Crambe crambe* | *Dysidea avara* | *Leptopsammia*  *pruvoti* | *Parazoanthus axinellae “slender”* | *Parazoanthus axinellae “stocky”* |
| --- | --- | --- | --- | --- | --- | --- | --- | --- | --- |
| *A. acaule* | *** |  |  |  |  |  |  |  |  |
| *A. damicornis* | n.s | *** |  |  |  |  |  |  |  |
| *A. polypoides* | ** | n.s | ** |  |  |  |  |  |  |
| *C. crambe* | *** | n.s | ** | n.s |  |  |  |  |  |
| *D. avara* | *** | ** | *** | ** | *** |  |  |  |  |
| *L. pruvoti* | n.s | *** | n.s | ** | ** | *** |  |  |  |
| *P. axinellae “slender”* | * | * | n.s | n.s | n.s | *** | n.s |  |  |
| *P. axinellae “stocky”* | *** | * | *** | * | * | * | *** | ** |  |
| *P. ficiformis* | *** | n.s | *** | * | *** | n.s | *** | *** | n.s |

| **28 ºC** | *Agelas oroides* | *Alcyonium acaule* | *Axinella damicornis* | *Axinella polypoides* | *Crambe crambe* | *Dysidea avara* | *Leptopsammia*  *pruvoti* | *Parazoanthus axinellae “slender”* | *Parazoanthus axinellae “stocky”* |
| --- | --- | --- | --- | --- | --- | --- | --- | --- | --- |
| *A. acaule* | *** |  |  |  |  |  |  |  |  |
| *A. damicornis* | * | *** |  |  |  |  |  |  |  |
| *A. polypoides* | ** | *** | n.s |  |  |  |  |  |  |
| *C. crambe* | *** | n.s | *** | *** |  |  |  |  |  |
| *D. avara* | *** | *** | *** | *** | * |  |  |  |  |
| *L. pruvoti* | n.s | *** | n.s | n.s | *** | *** |  |  |  |
| *P. axinellae “slender”* | *** | n.s | n.s | n.s | ** | *** | * |  |  |
| *P. axinellae “stocky”* | *** | * | *** | *** | n.s | * | *** | ** |  |
| *P. ficiformis* | *** | n.s | *** | *** | n.s | *** | *** | n.s | n.s |

| **29 ºC** | *Agelas oroides* | *Alcyonium acaule* | *Axinella damicornis* | *Axinella polypoides* | *Crambe crambe* | *Dysidea avara* | *Leptopsammia*  *pruvoti* | *Parazoanthus axinellae “slender”* | *Parazoanthus axinellae “stocky”* |
| --- | --- | --- | --- | --- | --- | --- | --- | --- | --- |
| *A. acaule* | *** |  |  |  |  |  |  |  |  |
| *A. damicornis* | *** | *** |  |  |  |  |  |  |  |
| *A. polypoides* | *** | *** | n.s |  |  |  |  |  |  |
| *C. crambe* | *** | n.s | ** | ** |  |  |  |  |  |
| *D. avara* | *** | *** | *** | *** | ** |  |  |  |  |
| *L. pruvoti* | ** | *** | ** | n.s | *** | *** |  |  |  |
| *P. axinellae “slender”* | ** | *** | * | n.s | *** | *** | n.s |  |  |
| *P. axinellae “stocky”* | *** | n.s | *** | *** | * | n.s | *** | *** |  |
| *P. ficiformis* | *** | ** | *** | *** | ** | n.s | *** | *** | n.s |

**References**

Cachet, N., Genta-Jouve, G., Ivanisevic, J., Chevaldonné, P., Sinniger, F., Culioli, G., … Thomas, O. P. (2015). Metabolomic profiling reveals deep chemical divergence between two morphotypes of the zoanthid *Parazoanthus axinellae*. *Scientific Reports* 5:8282. <https://doi.org/10.1038/srep08282>

Casas-Güell, E., Teixidó, N., Garrabou, J. & Cebrian, E. (2015). Structure and biodiverstiy of coralligenous assemblages over broad spatial and temporal scales. *Marine Biology*, 162, 901–912. <https://doi.org/10.1007/s00227-015-2635-7>

Cerrano, C., Bavestrello, G., Bianchi, C. N., Cattaneo-vietti, R., Bava, S., Morganti, C., … Sponga, F. (2000). A catastrophic mass-mortality episode of gorgonians and other organisms in the Ligurian Sea (northwestern Mediterranean), summer 1999. *Ecology Letters*, 3, 284–293. <https://doi.org/10.1046/j.1461-0248.2000.00152.x>

Cerrano, C., Magnino, G., Sarà, A., Bavestrello, G. & Gaino, E. (2001). Necrosis in a population of *Petrosia ficiformis* (Poriera, Demospogiae) in relation with environmental stress. *Italian Journal of Zoology*, 68: 131-136. <https://doi.org/10.1080/11250000109356397>

Cerrano, C., Totti, C., Sponga, F. & Bavestrello, G. (2006). Summer disease in *Parazoanthus axinellae* (Schmidt, 1862) (Cnidaria, Zoanthidea). Italian Journal of Zoology, 73 (4), 355-361. <https://doi.org/10.1080/11250000600911675>

Ferretti, C., Vacca, S., De Ciucis, C., Marengo, B., Duckworth, A. R., Manconi, R., … Domenicotti, C. (2009). Growth dynamics and bioactivity variation of the Mediterranean demosponges *Agelas oroides* (Agelasida, Agelasidae) and *Petrosia ficiformis* (Haplosclerida, Petrosiidae). *Marine Ecology*. 30: 1–10. <https://doi.org/10.1111/j.1439-0485.2008.00278.x>

Garrabou, J., Coma, R., Bensoussan, N., Bally, M., Chevaldonne, P., Cigliano, M., … Cerrano, C. (2009). Mass mortality in Northwestern Mediterranean rocky benthic communities: effects of the 2003 heat wave. *Global Change Biology*, 15, 1090–1103. <https://doi.org/10.1111/j.1365-2486.2008.01823.x>

Gili, J. M., Garcia, A. & Colomer, P. L. (1984). Els cnidaris bentònics de les Illes Medes. In: Ros J.D., Olivella I., Gili J.M. (eds) *Els sistemes naturals de les illes Medes. Arxius Sec Ciències Institut d’Estudis Catalans, Barcelona* 73: 407-427

Kaplan, E. L. & Meier, P. (1958). Nonparametric estimation from incomplete observations. *Journal of American Statistical Association*. 53: 457–481.

Linares, C., Ballesteros, E., Verdura, J., Aspillaga, E., Capdevila, P., Coma, R., … Cebrian, E. (2017). Efectos del cambio climático sobre la gorgonia Paramuricea clavata y el coralígeno asociado en el Parque Nacional Marítimo-Terrestre del archipiélago de Cabrera. In: Proyectos de investigación en Parques nacionales: convocatoria 2012-2015 (ed In: Proyectos de investigación en Parques Nacionales: convocatoria 2012-2015 (P. Amengual ed). OA de PN), pp. 45–67

López-Legentil, S., Ruchty, M., Domenech, A. & Turon, X. (2005). Life cycles and growth rates of two morphotypes of *Cystodytes* (Ascideacea) in the western Mediterranean. *Marine Ecology Progress Series*. 296: 219-228. <https://doi.org/10.3354/meps296219>

Maldonado, M. & Riesgo, A. (2009). Gametogenesis, embryogenesis, and larval features of the oviparous sponge *Petrosia ficiformis* (Haplosclerida, Demospongiae). *Marine Biology*. 156: 2181-2197. <https://doi.org/10.1007/s00227-009-1248-4>

Mantel, N. (1966). Evaluation of survival data and two new rank order statistics arising in its consideration. *Cancer Chemotherapy Reports*. 50: 163–70

Parravicini, V., Guidetti, P., Morri, C., Montefalcone, M., Donato, M. & Bianchi, C. N. (2010). Consequences of sea water temperature anomalies on a Mediterranean submarine cave ecosystem. *Estuarine Coastal and Shelf Science*, 86, 276-282. [https://doi.org/ 10.1016/j.ecss.2009.11.004](https://doi.org/%2010.1016/j.ecss.2009.11.004)

Perez, T., Garrabou, J., Sartoretto, S., Harmelin, J. G., Francour, P. & Vacelet, J. (2000) Mortalité massive d’invertébrés marins: un événemnt sans précédent en Méditerranée nord-occidentale. *Comptes Rendus de l´ Académie des Sciencies Series III Life Sciences,* 323:853–865. <https://doi.org/10.1016/S0764-4469(00)01237-3>

Previati, M., Palma, M., Bavestrello, G., Falugi, C. & Cerrano, C. (2010). Reproductive biology of *Parazoanthus axinellae* (Schmidt, 1862) and *Savalia savaglia* (Bertoloni, 1819) (Cnidaria, Zoantharia) from the NW Mediterranean coast. *Marine Ecology.* 31: 555-565. <https://doi.org/10.1111/j.1439-0485.2010.00390.x>

Uriz, M. J. (1986). Clave de la identificación de las esponjas más frecuentes de la Península Ibérica. Misc Zool. 10: 7-22

Uriz, M. J., Rosell, D. & Martin, D. (1992). The sponge population of the Cabrera Archipelago (Balearic Islands): characteristics, distribution and abundances of the most representative species. PSZN1. *Marine Ecology*. 2: 101-117

Van Soest, R. W. M. (2001). Porifera, in: Costello, M.J. *et al.* (Ed.) (2001). European register of marine species: a check-list of the marine species in Europe and a bibliography of guides to their identification. Collection Patrimoines Naturels 50: 85-103.

Zibrowius, H. (1980). Les Scléractiniaires de la Méditerranée et de l'Atlantique nord-oriental. Mem. Ist Ocenogr. Monaco, 11: 227 pp
